# Bi_2_WO_6_/C-Dots/TiO_2_: A Novel Z-Scheme Photocatalyst for the Degradation of Fluoroquinolone Levofloxacin from Aqueous Medium

**DOI:** 10.3390/nano10050910

**Published:** 2020-05-08

**Authors:** Shelja Sharma, Alex O. Ibhadon, M. Grazia Francesconi, Surinder Kumar Mehta, Sasikumar Elumalai, Sushil Kumar Kansal, Ahmad Umar, Sotirios Baskoutas

**Affiliations:** 1 Chemical Engineering Division, Centre of Innovative and Applied Bioprocessing, Mohali-140306, India; sshhelja@yahoo.in (S.S.); sasikumar@ciab.res.in (S.E.); 2 Department of Chemical Engineering, The University of Hull, Hull HU6 7RX, UK; a.o.ibhadon@hull.ac.uk; 3 Department of Chemistry, University of Hull, Hull HU6 7RX, UK; m.g.francesconi@hull.ac.uk; 4 Department of Chemistry and Centre of Advanced Studies, Panjab University, Chandigarh 160014, India; skmehta@pu.ac.in; 5 Dr. S.S. Bhatnagar University Institute of Chemical Engineering and Technology, Panjab University, Chandigarh 160014, India; 6 Department of Chemistry, Faculty of Science and Arts, Promising Centre for Sensors and Electronic Devices (PCSED), Najran University, Najran 11001, Saudi Arabia; 7 Department of Materials Science, University of Patras, 26504 Rio Achaia, Patras, Greece; bask@upatras.gr

**Keywords:** Bi_2_WO_6_/C-dots/TiO_2_, Z-scheme, photocatalysis, levofloxacin, solar irradiation

## Abstract

Photocatalytic materials and semiconductors of appropriate structural and morphological architectures as well as energy band gaps are materials needed for mitigating current environmental problems, as these materials have the ability to exploit the full spectrum of solar light in several applications. Thus, constructing a Z-scheme heterojunction is an ideal approach to overcoming the limitations of a single component or traditional heterogeneous catalysts for the competent removal of organic chemicals present in wastewater, to mention just one of the areas of application. A Z-scheme catalyst possesses many attributes, including enhanced light-harvesting capacity, strong redox ability and different oxidation and reduction positions. In the present work, a novel ternary Z-scheme photocatalyst, i.e., Bi_2_WO_6_/C-dots/TiO_2,_ has been prepared by a facile chemical wet technique. The prepared solar light-driven Z-scheme composite was characterized by many analytical and spectroscopic practices, including powder X-ray diffraction (XRD), field emission scanning electron microscopy (FE-SEM), N_2_ adsorption–desorption isotherm, Fourier-transform infrared spectroscopy (FT-IR), photoluminescence (PL) and UV-vis diffuse reflectance spectroscopy (DRS). The photocatalytic activity of the Bi_2_WO_6_/C-dots/TiO_2_ composite was evaluated by studying the degradation of fluoroquinolone drug, levofloxacin under solar light irradiation. Almost complete (99%) decomposition of the levofloxacin drug was observed in 90 min of sunlight irradiation. The effect of catalyst loading, initial substrate concentration and pH of the reaction was also optimized. The photocatalytic activity of the prepared catalyst was also compared with that of bare Bi_2_WO_6_, TiO_2_ and TiO_2_/C-dots under optimized conditions. Scavenger radical trap studies and terephthalic acid (TPA) fluorescence technique were done to understand the role of the photo-induced active radical ions that witnessed the decomposition of levofloxacin. Based on these studies, the plausible degradation trail of levofloxacin was proposed and was further supported by LC-MS analysis.

## 1. Introduction

The occurrence of pharmaceuticals and personal care products (PPCPs) in environmental samples has been observed over the past few decades and these compounds are now considered as contaminants of emerging concern, as they are biologically active even at low concentrations [1,2,3,4,5]. Pharmaceuticals have appeared as rapidly growing environmental contaminants and are one of the major concerns of national public health experts [6,7]. Hence, these substances have been called “pseudo-persistent” pollutants due to their unregulated and continuous discharge directly or indirectly to water systems via several ways [8,9]. A large amount of these compounds enters water bodies primarily from wastewater treatment plant effluents and other sources, such as hospital discharge, inappropriate disposal by the manufacturer and from expired and unused drugs [10,11,12]. Local release of pharmaceutical drugs may cause problems in aquatic environments since they are active at low concentrations ranging from ng/L–µg/L [13,14]. Levofloxacin is such an example of a broad-spectrum fluoroquinolone antibiotic that is widely used as an antibacterial agent, having bactericidal activity [15,16]. The high consumption of fluoroquinolone drug and its poor disposal into water sources has resulted in a generation of more toxic and resistant fecal bacteria [17]. Conventional wastewater treatment plants are not designed for the complete elimination of these compounds owing to their resistance to biodegradation. The photocatalysis technique has emerged as a promising technology and has garnered increased research attention [18,19,20,21]. Photocatalysts with broad absorption range, narrow band gap, high stability, efficient charge separation, high redox abilities can directly convert solar energy into storable energy compounds and degrade hazardous organic pollutants into least toxic compounds [22,23,24,25,26,27]. However, a single photocatalyst cannot possess all the ideal characteristics; for example, wide absorption range and strong redox abilities are difficult to achieve simultaneously in a single photocatalyst [28]. These properties can be achieved by designing appropriate photocatalytic systems. In a heterojunction type photocatalytic system, two photocatalysts with dissimilar band gap values are involved, the photo-induced electrons from the conduction band (CB) of one photocatalyst (PC1) migrate to the CB of another photocatalyst (PC2), and photo-induced holes also migrate from the valence band (VB) of PC2 to VB of PC1, thereby leading to increased charge separation and reduced recombination of charge carriers. However, the redox capabilities of both photocatalysts are neglected as the oxidation and reduction processes occur on the semiconductors with the lower oxidation and reduction potentials, respectively. Hence, even if this heterogeneous photocatalytic system can hinder the recombination of photogenerated charge carriers, it cannot possess efficient charge separation and the strong redox ability simultaneously [29,30]. To address these limitations, an investigation of more efficient photocatalytic systems is required.

The artificial Z-scheme photocatalytic system can overcome the drawbacks associated with a single component and heterojunction type photocatalyst systems. It mimics the photosynthesis process that includes a two-step photoexcitation. Like a heterojunction-type photocatalytic system, it can also efficiently overcome the recombination of photogenerated charge carriers, although in this case, strong redox abilities can also be achieved by combining two narrow band gap semiconductors. In the typical mechanism, the photogenerated electrons on CB of PCI do not migrate to PCII, making PCI electron-rich region and thereby, obstructing the photo-oxidation of PCI and simultaneously making PCII a hole rich region which can cover PCII from photo-oxidation [31,32]. 

Carbon quantum dots (C-dots) are quite attractive in the field of photocatalysis, as these can act as an electron mediator, a photosensitizer, and a spectral converter in photocatalytic processes. In particular, C-dots show up-conversion photoluminescence (PL), an optical phenomenon in which materials emit light of shorter wavelength than the excitation source. Such feature can significantly expand the solar light absorption of wide band gap semiconductors into the visible region and the near infrared region. Moreover, photo-induced C-dots serve as both excellent electron donors and acceptors, thereby resulting in effective charge separation of electrons and holes. Considering the remarkable properties of C-dots, the combination of C-dots and other metal oxide-based semiconductors may be regarded as an ideal strategy for the designing of high performance photocatalyst for enhanced catalytic activity.

Therefore, in the present work, a ternary Z-scheme photocatalyst, (Bi_2_WO_6_/C-dots/TiO_2_), was synthesized by a facile surfactant free hydrothermal method. The synthesized catalyst was characterized by analytical and spectroscopic techniques, and further, its photocatalytic activity was tested against the decomposition of the antibiotic levofloxacin under sunlight.

## 2. Materials and Methods

L-ascorbic acid (C_6_H_8_O_6_, <99.0%), bismuth (III) nitrate pentahydrate (Bi(NO_3_)_3_·5H_2_O, 98.0%), sodium tungsten oxide dihydrate (Na_2_WO_4_·2H_2_O, 95.0%) and ethanol (C_2_H_5_OH) were bought from Alfa Aesar, United Kingdom. Terephthalic acid (TPA) (C_8_H_6_O_4_, 99.0%), sodium hydroxide (NaOH, 99.0%), formic acid (HCOOH, 99.0%), potassium iodide (KI, 99.0%) and titanium isopropoxide (TTIP, <99.0%) and were purchased from Sigma-Aldrich, India. Fluoroquinolone drug levofloxacin was procured from Saurav Chemicals, Derabassi, India. Deionized water was used for all experiments.

### 2.1. Preparation of the Catalysts

#### 2.1.1. Synthesis of Bi_2_WO_6_

Bi_2_WO_6_ was synthesized by the hydrothermal method. In a brief process, 0.006 mol (1.455 g) of bismuth nitrate pentahydrate (Bi(NO_3_)_3_·5H_2_O) was solubilized in 37.5 mL of water, labeled as solution A. 0.003 mol (0.5 g) of sodium tungstate dihydrate (Na_2_(WO_4_) 2H_2_O) was dissolved in 12.5 mL of ethylene glycol and labeled as solution B. The latter was introduced into solution A under sonication with dropwise addition. The resultant sol was stirred for an hour and then transferred to a 75 mL Teflon-lined stainless-steel autoclave for hydrothermal treatment at 160 °C for 25 h. After the reaction, the precipitates were collected via centrifugation and washed with ethanol and water thoroughly and dried at 50 °C overnight.

#### 2.1.2. Synthesis of TiO_2_/C-Dots

TiO_2_ was synthesized by hydrothermal method with slight modifications from previous work [22]. The hydrothermal approach was used to synthesize uniform and dispersed C-dots nanoparticles with controllable shapes and sizes and high stability. In this procedure, 6 mL of TTIP was added to 10 mL of ethanol and the mixture was stirred for 30 min and designated as solution C. Furthermore, the equimolar concentrations of water and ethanol solution (50 mL) named as solution D was prepared. Solution D was introduced into solution C slowly with constant stirring for 30 min. Immediate turbidity was seen in the solution after the addition. The turbid solution was then placed in Teflon lined stainless steel autoclave. Meanwhile, L-ascorbic acid (0.5 g) was dissolved into 50 mL of ethanol and kept under stirring and then it was transferred into the Teflon autoclave for hydrothermal treatment at 180 °C for 4 h. The obtained precipitates were separated by centrifugation after the completion of the hydrothermal reaction. The precipitates were carefully washed with the water and ethanol and kept for drying in an oven at 60 °C. The color of powder changed from clear white to pale white, specifying the successful synthesis of TiO_2_/C-dots.

#### 2.1.3. Synthesis of Bi_2_WO_6_/C-Dots/TiO_2_

Bismuth tungstate (0.5 g) and TiO_2_/C-dots (0.25 g) were dispersed in 25 mL of ethanol and kept under magnetic stirring overnight. The precipitates collected were then washed, filtered and dried at 50 °C overnight.

### 2.2. Characterizations of Prepared Catalysts

The crystallinity and structure analysis of the catalysts were determined by Powder X-ray diffraction (PANalytical Empyrean X-ray diffractometer, united Kingdom) with Cu K_α1_ radiation. Results were recorded using the instruments inbuilt software. The XRD patterns obtained were analyzed using PANalytical Highscore Plus. Fourier Transformation Infra-red of the powder samples were recorded on spectrometer (Thermo Scientific Nicolet iS5 FTIR, United Kingdom) (wavenumber range: 600–4000 cm^−1^). The surface morphology of the samples was analyzed using the field-emission electron microscope (FE-SEM; Hitachi-SU8010, India), operating at the voltage of 15 kV equipped with energy dispersive spectroscopy (EDS; Bruker XFlash, India). The thermogravimetric analysis (TGA) of the catalysts was performed on Simultaneous Thermal Analyzer (STA; Perkin Elmer, India) 8000 in the temperature range 30 °C to 800 °C. The fluorescence spectra of the samples were recorded on spectrophotometer (Cary Eclipse Agilent Technologies, India). The UV-vis DRS spectra of the photocatalysts were obtained from spectrophotometer (Shimadzu UV-2600, India) with barium sulphate taken as a reference. The nitrogen adsorption and desorption isotherms of the prepared catalyst were measured using surface area analyzer (BELSORP, India) prior to the analysis, the samples were degassed at 100 °C for 24 h. The pore size distribution curve, total pore volume and mean pore diameter were determined from the desorption branch of Barrett–Joyner–Halenda (BJH) model. The absorbance of the samples was recorded on the Shimadzu UV-Vis spectrophotometer (UV-2600). Total organic carbon (TOC) was recorded on TOC (TOC-L CPN Shimadzu, India) analyzer. The liquid chromatography–mass spectroscopy (LC-MS) of the drug samples was analyzed on spectrophotometer (Agilent Technologies 6120 Quadrupole, India) to determine the degradation products formed and other intermediates.

### 2.3. Photocatalytic Experiments

The photocatalytic experiments were carried out using antibiotic drug levofloxacin as a target pollutant and its degradation was observed under direct sunlight irradiation. The solar experiments were carried out under direct sunlight in a cylindrical slurry batch reactor with an average intensity of 70 Klux. In a typical photocatalytic test, different amounts of the prepared photocatalyst were introduced in levofloxacin solution (100 mL, 10 mg/L) and kept under stirring in the dark for 30 min for the achievement of adsorption–desorption process. The reaction solution was then subjected to solar light irradiation. The samples (2 mL) were drawn from the reaction at set time periods and made to pass via 0.22 µm Millex syringe filter. Other process parameters, i.e., catalyst dosage, pH and initial pollutant concentration, were studied for the photocatalytic experiments. The % degradation can be determined from the equation given below:

(1) $$\%\;\mathrm{degradation}\;=\frac{C_{0}-C}{C}\times 100$$

where *C*_0_ and *C* are the initial drug concentration and concentration of drug at time *t*.

### 2.4. Investigation of Function of Active Radical Ions in the Decomposition of Levofloxacin under Sunlight Illumination

The generation of hydroxyl radicals from the sunlight illuminated Bi_2_WO_6_/C-dots/TiO_2_ was examined using a TPA fluorescence technique. The levofloxacin drug solution was replaced with 100 mL of 0.5 mM TPA solution in 2 mM sodium hydroxide. The optimized dose of photocatalyst was added into the TPA solution and illuminated by direct sunlight under constant stirring and the samples were drawn from the respective solutions at pre-designed intervals. The aliquots were then made to pass through filter and the corresponding fluorescence spectra were obtained from PL spectrophotometer at λ_ex_ = 315 nm. Furthermore, scavenger experiments were implemented to investigate the occurrence and character of active radical ions in the photocatalytic system. Potassium iodide (KI) and formic acid (HCOOH) of concentrations 0.01 M, were served as scavenger for holes (h^+^) and electrons (e^−^) in the reaction system. The quenchers were dispersed into the levofloxacin drug solution before adding the catalyst and solar light illumination, maintaining other optimized parameters constant, i.e., drug concentration, catalyst dose and pH of drug solution.

## 3. Results and Discussion

### 3.1. Characterization of the Synthesized Catalysts 

**Figure 1:**
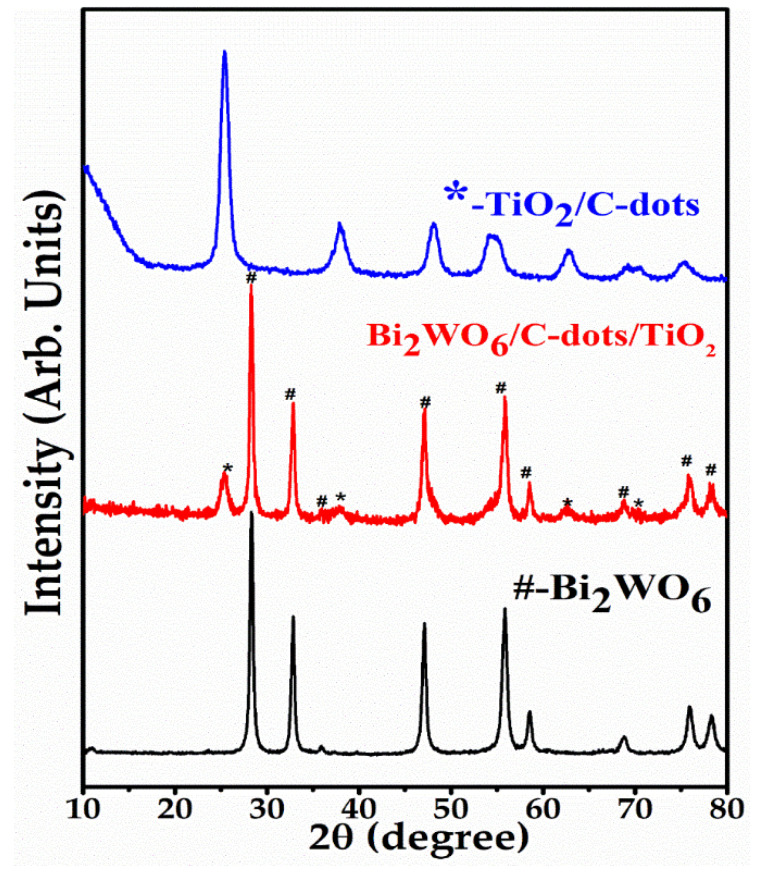
XRD spectra of Bi_2_WO_6_ (#), TiO_2_/C-dots (*), and Bi_2_WO_6_/C-dots/TiO_2_.

The crystallinity, structural characteristics and purity analysis were studied by the powder XRD technique. The powder XRD patterns of Bi_2_WO_6_, TiO_2_/C-dots and Bi_2_WO_6_/C-dots/TiO_2_ are displayed in Figure 1. In the case of TiO_2_/C-dots, the well-known and sharp peaks at 2*θ* = 25.4°, 38.10°, 48.05°, 53.80°, 55.11° and 62.81° were observed, which correspond to the pure anatase phase of TiO_2_, and the results were found to be consistent with JCPDS card no. 21-1272 [33,34]. It is clear from the figure that no distinctive peak for C-dots around 20°–24° was observed in the pattern, which might be due to the reduced concentration of C-dots. The values of lattice constants “a” and “c” were 3.78 Å and 9.44 Å, calculated from tetragonal crystal. In case of Bi_2_WO_6_, the diffraction peaks centered at 2*θ* = 28.30°, 32.81°, 35.90°, 47.12°, 55.81°, 58.52°, 68.90°, 76.0° and 78.3° are attributed to the orthorhombic phase of Bi_2_WO_6_, and are in agreement with JCPDS card No. 39-0256 [35,36,37,38]. The values of lattice constants of Bi_2_WO_6_ were computed to be 5.456 Å (a), 5.454 Å (b) and 16.455 Å (c). In the XRD pattern of Bi_2_WO_6_/C-dots/TiO_2_, all the peaks belonging to Bi_2_WO_6_ and TiO_2_/C-dots were present, thereby confirming the successful synthesis of Z-scheme catalyst. Narrow and sharp diffraction patterns confirmed the crystalline nature of the composite. The average crystallite size of the Z-scheme photocatalyst was computed from Debye-Scherrer’s formula as given below:

D = 0.94λ/βcosθ(2)

where D is the crystallite size (average), λ is the wavelength of X-ray radiation, β is the broadening of the diffraction line assessed at full-width half maximum value (FWHM) and θ is the Bragg’s angle. The crystallite size of the Z-scheme catalyst (Bi_2_WO_6_/C-dots/TiO_2_) was computed to be 34.29 nm.

**Figure 2:**
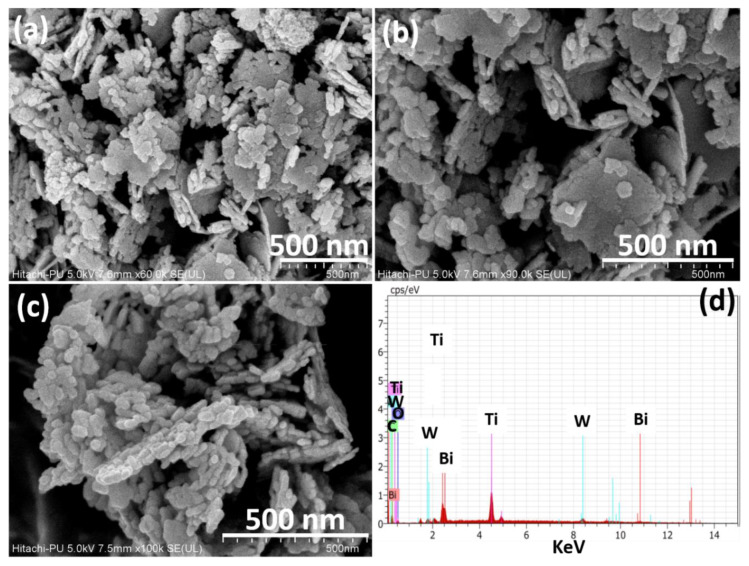
(**a**–**c**) FESEM images of Bi_2_WO_6_/C-dots/TiO_2_ at different magnifications. (**d**) EDX of Bi_2_WO_6_/C-dots/TiO_2._

The morphologies of Bi_2_WO_6_, TiO_2_/C-dots and Bi_2_WO_6_/C-dots/TiO_2_ were determined using FESEM provided with EDS (Figure 2). In Figure 2a, it can be seen that Bi_2_WO_6_ exhibited flakes like morphology of irregular size. The edges of flakes were found to be asymmetrical and uneven; however, the base and sides were found to be smooth (Figure 2b). The nanoflakes were grown in high density, and these were clustered together with each other in random fashion exhibiting high aspect ratio (Figure 2c), whereas the nanoparticles of the TiO_2_/C-dots were embedded at the surface of flakes. EDS spectrum of Bi_2_WO_6_/C-dots/TiO_2_ displayed the presence of bismuth (Bi), titanium (Ti), tungstate (W), carbon (C) and oxygen (O) in the photocatalyst (Figure 2d). The morphologies of Bi_2_WO_6_ and TiO_2_/C-dots were also studied for the comparison, and these are provided in Supplementary Information. TiO_2_/C-dots consisted of nanoparticles with sizes less than 10 nm, and Bi_2_WO_6_ possessed a nanoflake-like morphology, where the flakes were found to be highly agglomerated (Figures S1 and S2). The EDS spectrum of Bi_2_WO_6_ is given in the Supplementary Information (Figure S1) and its elemental composition is depicted in Table S1.

**Figure 3:**
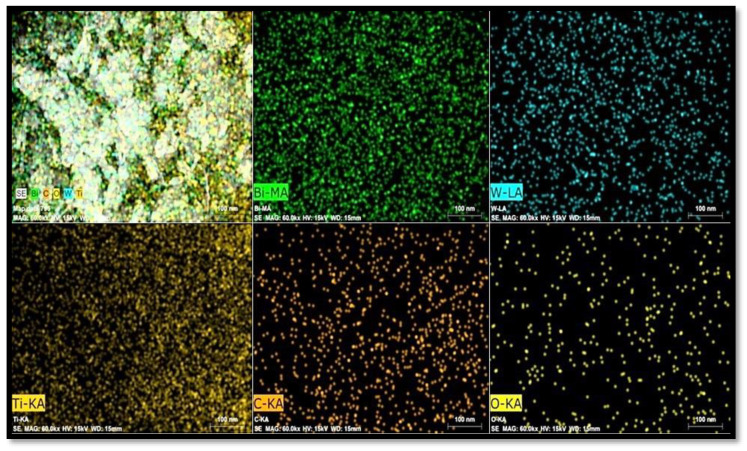
Elemental mapping of Bi_2_WO_6_/C-dots/TiO_2_ showing different colors for Bi, W, Ti, C and O.

Furthermore, the synthesis of Z-scheme catalyst (Bi_2_WO_6_/C-dots/TiO_2_) was also confirmed by elemental mapping, as shown in Figure 3. The mapping outcomes concluded that the elements concerned, i.e., Bi, Ti, W and C and O, were present in various proportions and quantities in the catalyst. The elemental composition of the sample from the EDS spectrum is presented in Table 1.

**Figure 4:**
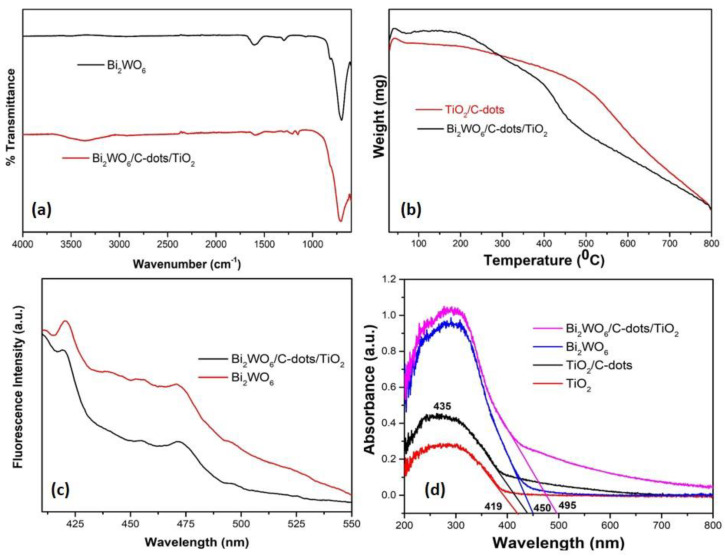
(**a**) FTIR analysis of Bi_2_WO_6_ and Bi_2_WO_6_/C-dots/TiO_2_. (**b**) Thermogravimetric analysis of TiO_2_/C-dots and Bi_2_WO_6_/C-dots/TiO_2_. (**c**) PL spectra of Bi_2_WO_6_ and Bi_2_WO_6_/C-dots/TiO_2_ at λ_ex_ = 360 nm. (**d**) UV-vis DRS analysis of TiO_2_, TiO_2_/C-dots, Bi_2_WO_6_, and Bi_2_WO_6_/C-dots/TiO_2_.

FTIR spectroscopy revealed the presence of functional groups and other chemical composition of catalyst as shown in Figure 4a. The broad absorption curve in the region 3000–3400 cm^−1^ and the sharp peak at 1620 cm^−1^ might be related to the bending and stretching modes of adsorbed water on the surface of the catalyst [39,40]. The distinctive bands between 600 and 1000 cm^−1^ were attributed to the W-O bond in terms of stretching and bridging modes. For instance, W-O stretching vibration was positioned at 682 cm^−1^ and W-O-W bending vibrations modes were centered at 821 cm^−1^ and 1290 cm^−1^ [41,42]. Thermogravimetric analysis of Bi_2_WO_6_/C-dots/TiO_2_ and TiO_2_/C-dots presented the thermal properties of the synthesized catalysts (Figure 4b). It was noticed that TiO_2_/C-dots showed 22.8% weight loss (initial weight: 3.506 g, weight loss: 0.8 g), whereas Bi_2_WO_6_/C-dots/TiO_2_ exhibited only 7.73% weight loss (initial weight: 4.306 g, weight loss: 0.33 g) at temperatures ranging from 30 °C to 800 °C. The weight loss in both cases was because of the desorption of adsorbed water from the surface of the catalysts and degradation of oxygen-containing functional groups from the surface [43]. The Z-scheme nano-photocatalyst exhibited exceptionally high thermal stability throughout the whole temperature range.

The optical characteristics of the synthesized Z-scheme nano-photocatalyst were studied using the PL and UV-vis DRS. PL emission is directly related to the efficient separation of photo-induced charge transporters. PL spectra of Bi_2_WO_6_ and Bi_2_WO_6_/C-dots/ TiO_2_ were obtained at an excitation wavelength of 360 nm (Figure 4c). Bi_2_WO_6_ exhibited a sharp peak at 421 nm ascribed to the higher probabilities of the recombination of photo-induced charge transporters. Few PL emission bands in the wavelength area 450–500 nm were related to the blue-green emission band. On the other hand, in Bi_2_WO_6_/C-dots/TiO_2_, the PL intensity of sharp peak at 421 nm was also reduced, implying that there would be lesser recombination of photo-induced charge carriers. The outcome suggested that a greater number of induced charge carriers would be available to participate in the photocatalytic mechanism. The broad and minor peak at 525 nm is related to metal atom/crystal defects owing to oxygen vacancies [44,45]. The absorption properties and band gap of Bi_2_WO_6_ and Bi_2_WO_6_/C-dots/TiO_2_ were determined by UV-vis DRS (Figure 4d). The energy band gap of TiO_2_, TiO_2_/C-dots, Bi_2_WO_6_, and Bi_2_WO_6_/C-dots/TiO_2_ was calculated using the equation below:

(3) $$E_{g}=\frac{1240}{\lambda_{max}}$$

where *E_g_* is the calculated energy band gap (eV) and *λ_max_* is the observed wavelength calculated from the edge of the absorption curve by drawing a tangent to it. 

The maximum absorption edges of TiO_2_, TiO_2_/C-dots, Bi_2_WO_6_, and Bi_2_WO_6_/C-dots/TiO_2_ were observed at 419 nm, 435 nm, 450 nm and 495 nm, and the corresponding values of E_g_ were estimated to be 2.95 eV, 2.85 eV, 2.75 eV and 2.50 eV. The lower value of the E_g_ indicated the enhanced light absorption ability of the Bi_2_WO_6_/C-dots/TiO_2_ and similar for the photocatalytic degradation of levofloxacin by Bi_2_WO_6_/C-dots/TiO_2_ [44]. PL studies also supported the improved degradation of levofloxacin by Bi_2_WO_6_/C-dots/TiO_2_ under direct sunlight.

**Figure 5:**
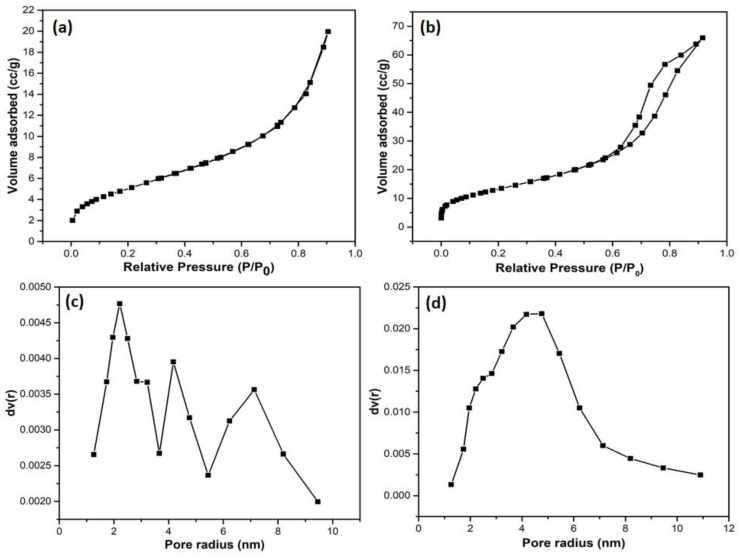
N_2_ adsorption–desorption isotherm of (**a**) Bi_2_WO_6_ and (**b**) Bi_2_WO_6_/C-dots/TiO_2_. BJH curve displaying pore size distribution of (**c**) Bi_2_WO_6_ and (**d**) Bi_2_WO_6_/C-dots/TiO_2_.

The specific surface area of the composite gives an idea about the accessibility of active sites on the surface of catalyst; the greater the specific surface area of the catalyst, the greater the improvement in the charge movement of photo-induced charge carriers, hence resulting in enhanced photocatalytic activity. Brunauer–Emmett–Teller (BET) results measure the surface area of porous materials. N_2_ adsorption–desorption isotherm and BJH pore size distribution curve of the synthesized Bi_2_WO_6_ and Bi_2_WO_6_/C-dots/TiO_2_ Z-scheme catalyst are shown in Figure 5. The hysteresis loop for both Bi_2_WO_6_ and Bi_2_WO_6_/C-dots/TiO_2_ Z-scheme catalyst was of type-IV isotherm, representing the typical mesoporous nature of nanostructures. The specific surface area of Bi_2_WO_6_ and Bi_2_WO_6_/C-dots/TiO_2_ was determined to be 18.364 m^2^/g and 48.8 m^2^/g, respectively, and total pore volume of Bi_2_WO_6_ and Bi_2_WO_6_/C-dots/TiO_2_ was computed to be 0.0309 cm^3^/g and 0.102 cm^3^/g, respectively. Hence, the increased specific surface area of the Z-scheme catalyst, i.e., Bi_2_WO_6_/C-dots/TiO_2_ over bare Bi_2_WO_6_, also promises enhanced degradation of levofloxacin drug under sunlight illumination.

### 3.2. Photocatalytic Activity of Z-Scheme Nano-Photocatalyst under Solar Light Illumination

**Figure 6:**
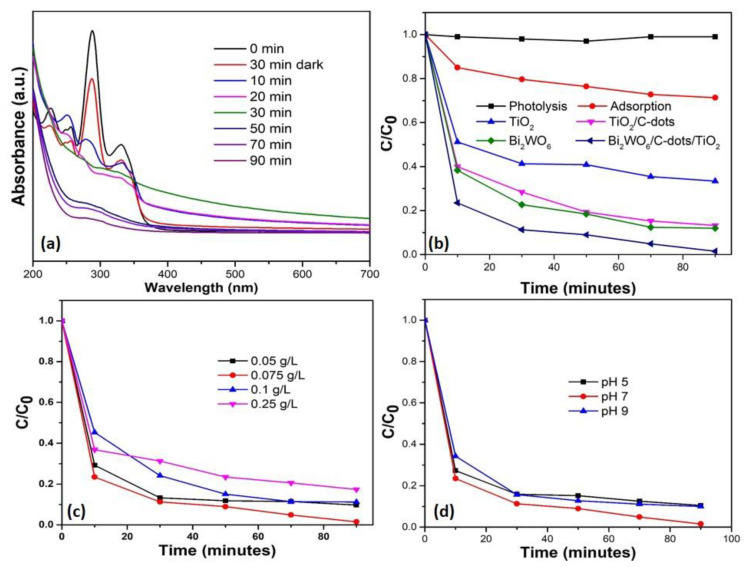
(**a**) Time-dependent UV-vis absorbance spectra of levofloxacin (10 mg/L, pH 7) under sunlight with Bi_2_WO_6_/C-dots/TiO_2_ (0.075 g/L). (**b**) Assessment of photolysis, adsorption and photocatalytic efficiency of Bi_2_WO_6_/C-dots/TiO_2_ with bare Bi_2_WO_6_, TiO_2_ and TiO_2_/C-dots (catalyst dose 0.075 g/L, pH 7, Conc. of drug 10 mg/L. (**c**) Effect of variation in catalyst dose (Bi_2_WO_6_/C-dots/TiO_2_) on photocatalytic degradation of drug. (**d**) Effect of change in pH of levofloxacin solution on photocatalytic behavior of Bi_2_WO_6_/C-dots/TiO_2_ (dose 0.075 g/L) under sunlight.

The photocatalytic efficiency of the synthesized catalysts was tested by observing the decomposition of levofloxacin (colorless pollutant). The fluoroquinolone drug levofloxacin belongs to the family of synthetic, broad-spectrum antibiotics, and showed an absorbance maximum at λ_max_ = 286 nm. The time-dependent UV-vis absorbance spectra of levofloxacin are demonstrated in Figure 6a. The absorbance of the levofloxacin drug solution (10 mg/L) tends to diminish with an increase in reaction time in the presence of Bi_2_WO_6_/C-dots/TiO_2_ under sunlight. The absolute degradation of the levofloxacin drug was achieved in 90 min of sunlight irradiation with 0.075 g/L dose of Z-scheme catalyst (Bi_2_WO_6_/C-dots/TiO_2_) with the drug solution at pH 7 (Figure 6a). Likewise, the degradation of the levofloxacin was monitored without the catalyst; however, under solar light illumination, the process is commonly known as photolysis, and there was no degradation observed in this case. Thus, photolysis proved incapable of the decomposition of levofloxacin (Figure 6b). However, 28% removal of the drug was obtained with the catalyst (0.075 g/L) in 90 min under dark conditions (Figure 6b). Therefore, it was revealed that the combination of light and the presence of a catalyst is mandatory for the efficient degradation of organic contaminants in aqueous medium. 

The photocatalytic efficiency of the Z-scheme catalyst was also compared with bare Bi_2_WO_6_, TiO_2_ and TiO_2_/C-dots under the same augmented operating conditions. Bi_2_WO_6_ exhibited 88.3% degradation, whereas TiO_2_ and TiO_2_/C-dots showed 66% and 87.24% degradation of levofloxacin with same catalyst dose after 90 min of photocatalytic reaction (Figure 6b). The similar percentage of degradation of both catalysts could be due to their almost equivalent E_g_ values, which were calculated from UV-vis DRS studies. 

The impact of catalyst dose was studied in order to optimize the amount of catalyst usage in the photocatalytic reaction. The catalyst (Bi_2_WO_6_/C-dots/TiO_2_) dose was varied from 0.05 g/L to 0.25 g/L, while maintaining the critical factors constant, i.e., the initial substrate concentration was kept at 10 mg/L and the pH of the drug solution was 7 (neutral). With the increase in the catalyst dose from 0.05 g/L to 0.075 g/L, the degradation of levofloxacin was observed to increase from 97% to 99% with Bi_2_WO_6_/C-dots/TiO_2_; however, the further rise in catalyst dose from 0.075 g/L to 0.1 g/L resulted in a decrease in the degradation of levofloxacin to 88.7%, and a further increase in catalyst dose to 0.25 g/L resulted in degradation dropping to 82.58%. This indicates that any further increments in catalyst dose will not lead to significant or enhanced degradation. The enhancement in photocatalytic activity with increased catalyst dose is attributed to the accessibility of a greater number of active sites on the surface of the Bi_2_WO_6_/C-dots/TiO_2_, and when the catalyst dose was increased above 0.075 g/L, the photocatalytic decomposition of levofloxacin decreased because of the shielding effect caused by nanoparticles at increased catalyst doses. This phenomenon explains the masking effect at the surface of the catalyst from illumination, hence obstructing the photons from reaching the substrate (levofloxacin). In other terms, the increased turbidity in the drug solution due to the presence of higher doses of catalyst led to less photon penetration and ultimately, unwanted scattering of photons [46]. The pH of the levofloxacin was also changed from 5 to 9 to optimize the reaction parameters while maintaining the catalyst dose (0.075 g/L) and initial drug concentration of drug solution (10 ppm) constant. The maximum degradation of the drug was achieved at neutral pH (7), i.e., 99%, and degradation of 89.4% and 90% was achieved at pH 5 and 9, respectively. The variation in the results might correspond to the surface charge of the catalyst. The effect of the pH on photocatalytic degradation of fluoroquinolones accounts for several factors, such as electrostatic contact between the photocatalyst surface, drug molecules, charged radicals and solvent molecules formed during the photocatalytic reaction process. The point of zero charge (pzc) for Bi_2_WO_6_ and TiO_2_ was reported to be 5.5 and 6.2 [47,48]. Therefore, at more acidic pH values, the surface of catalysts becomes positively charged, and at pH < 5.5 and 6.2, the drug behaves like cationic molecules. This leads to repulsion between catalyst and drug molecules due to the decrease in adsorption capacity of positive charged drug molecules on the catalyst surface. Additionally, levofloxacin drug in zwitterionic structure tends to generate three different ionic forms, which are completely dependent on its pH. Levofloxacin exists in zwitterionic form at 5.7–7.9 [49]. Furthermore, in alkaline solution, more hydroxyl radicals can be generated by sufficient hydroxide ions. The decrease in degradation efficiency at pH value 9 could also be due to the repulsion effect, thereby decreasing the adsorption of negatively charged drug molecules on the catalyst surface [50]. 

The photocatalytic degradation of levofloxacin was determined based on the extent of mineralization computed from the TOC values. The initial TOC value of levofloxacin drug solution was calculated to be 8.4 mg/L and the final TOC value of the drug solution after the photocatalytic treatment of 90 min was found to be 3.011 mg/L. The TOC results indicated that 64% mineralization of levofloxacin drug solution was observed with Bi_2_WO_6_/C-dots/TiO_2_ (0.075 g/L, pH 7 (drug solution) after 90 min of solar irradiation.

The initial drug concentration was also varied from 5 mg/L to 20 mg/L. Different concentrations of the drug solution were made by diluting the stock solution (20 mg/L). With the increase in levofloxacin drug concentration, the photocatalytic degradation of drug gradually decreased. The performance of the catalyst was predominantly dependent on the concentration of pollutant. Since the increased concentration of organic pollutant would lead to the reduced formation of oxidizing species and the active sites over the catalyst surface would be captured by the drug (substrate), hence resulting in lower degradation [51]. 

The recyclability of the Z-scheme catalyst was also determined in order to examine its chemical stability for up to three cycles. For each consecutive cycle, the catalyst was separated from the reaction mixture by centrifugation, washed thoroughly with a mixture of ethanol and distilled water (until the pH of supernatant is neutral) and dried in an oven at 80 °C. The percentages of degradation of levofloxacin for the three cycles were calculated to be 99%, 95% and 92%, respectively. The minor reduction in catalytic activity might be due to the leaching of bismuth and titanium ions and the loss of active sites during the degradation process. The ternary Z-scheme catalyst showed reasonable catalytic stability and can be used as a reusable catalyst, while thermal and mechanical stability could also be expected.

### 3.3. Plausible Mechanism for Levofloxacin Degradation with Z-Scheme Catalyst under Solar Light Irradiation

The CB and VB potentials of Bi_2_WO_6_ and TiO_2_ were computed from the calculations based on UV-DRS studies and also by employing the following equations:

E_VB_ = X_e_ − E_e_ + 0.5 E_g_ (4)

E_CB_ = E_VB_ − E_g_ (5)

where E_VB_ and E_CB_ represent the valence and conduction band edge potentials. E_e_ represents the energy of free electrons on hydrogen scale, i.e., 4.5 eV. E_g_ is calculated from UV-vis DRS studies for Bi_2_WO_6_, TiO_2_/C-dots and Bi_2_WO_6_/C-dots/TiO_2_ are 2.75 eV, 2.85 eV and 2.50 eV, respectively. Further, E_VB_ and E_CB_ of Bi_2_WO_6_ were calculated as 3.2eV and 0.59 eV, and for TiO_2_/C-dots, the corresponding values were 2.8 eV and -0.21 eV, respectively. Depending upon the similar energy band gap values of Bi_2_WO_6_ and TiO_2_, two probable charge transfer mechanisms can be derived: traditional type II (double charge transfer) and Z-scheme heterojunction (multiple charge transfer) mechanisms. If the transfer mechanism follows the double charge transfer theory, the photoinduced e^−^ on the CB of TiO_2_ would likely transfer to the CB of Bi_2_WO_6_ and the h^+^ on the VB of Bi_2_WO_6_ would migrate to the VB of TiO_2_, because the VB and CB potentials of Bi_2_WO_6_ are all lower than that of TiO_2_. However, the e^+^ on the CB of Bi_2_WO_6_ cannot reduce O_2_ to generate ^•^O^2−^ because the CB potential of Bi_2_WO_6_ (0.595 eV/vs. NHE) is lower than the standard redox potential of O_2_/^•^O^2−^ (−0.046 eV vs. NHE). Similarly, h^+^ on the VB of TiO_2_ (2.8 eV vs. NHE) also cannot efficiently oxidize H_2_O to yield ^•^OH owing to the almost equal redox potential of H_2_O/^•^OH with 2.73 eV vs. NHE. 

Therefore, in order to completely understand the degradation mechanism of antibiotic drug levofloxacin, multiple charge transfer mechanisms have to be followed. When Bi_2_WO_6_/C-dots/TiO_2_ is illuminated by solar light irradiation, the e^−^ on CB of Bi_2_WO_6_ and h^+^ on the VB of TiO_2_ recombine with themselves to form an inner heterojunction; nevertheless, the separated e^−^ and h^+^ aggregate on CB of TiO_2_ and VB of Bi_2_WO_6_, respectively. Meanwhile, C-dots on the surface of TiO_2_ are accepted as an electron reservoir. Owing to the upconversion PL of C-dots, longer wavelength light could be converted into 350–650 nm light (shorter wavelength). Then, Bi_2_WO_6_ and TiO_2_ are capable of consuming the upconverted UV (from C-dots) and visible light, and hence the e^−^-h^+^ pairs are produced on the surface of Bi_2_WO_6_ and TiO_2_. Moreover, due to the Z-scheme heterojunction between Bi_2_WO_6_ and TiO_2_, photoinduced e^−^ in the CB of Bi_2_WO_6_ moved quickly into the VB of TiO_2_ and paired with the h^+^, thereby leading to the buildup of e^−^ in the CB of TiO_2_ and h^+^ in the VB of Bi_2_WO_6_. Additionally, the increased number of e^−^ in the TiO_2_ could therefore be moved and gathered on the surface of C-dots. Afterward, the e^−^ reacted with the adsorbed O_2_ to form superoxide radicals (O_2•_^−^). In the meantime, the h^+^ in the VB of Bi_2_WO_6_ was able to oxidize OH^−^/H_2_O to form ^•^OH or participate in the degradation of organic pollutants [52,53]. Therefore, it is considered that the Bi_2_WO_6_/C-dots/TiO_2_ composite could follow the Z-scheme charge transfer mechanism. 

**Figure 7:**
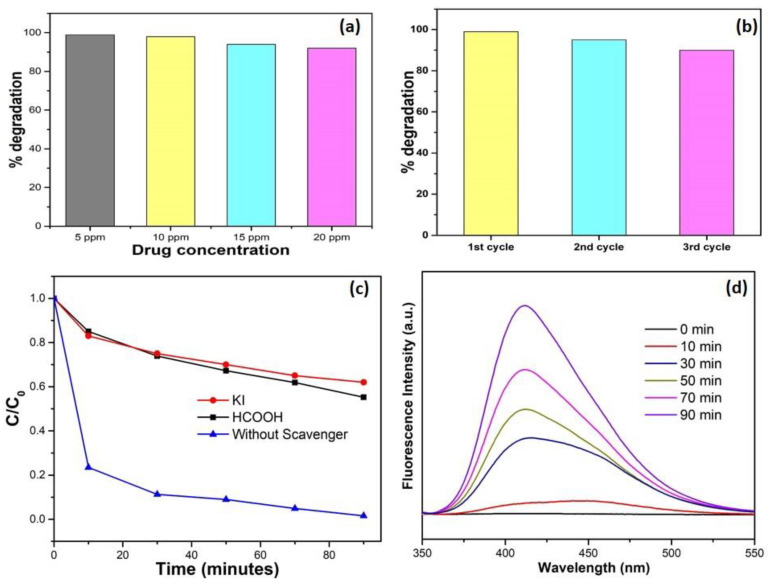
(**a**) Influence of initial concentration of drug on the degradation of levofloxacin. (**b**) No. of consecutive runs depicting recyclability of Bi_2_WO_6_/C-dots/TiO_2_. (**c**) Influence of scavengers on the photocatalytic activity of catalyst (drug conc. 10 ppm, pH 7, catalyst (Bi_2_WO_6_/C-dots/TiO_2_) dosage 0.075 g/L). (**d**) PL spectra of TPA in presence of catalyst (Bi_2_WO_6_/C-dots/TiO_2_) at λ_ex_ = 315 nm.

**Figure 8:**
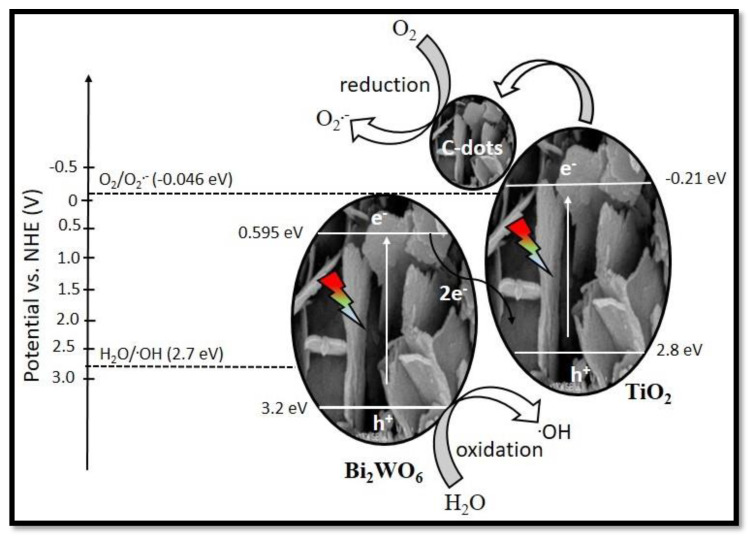
Plausible mechanism of photocatalytic degradation of levofloxacin with Z-scheme photocatalyst.

Scavenger radical studies also confirmed the formation of holes and electrons in the photocatalytic reaction mechanism. The photocatalytic degradation of levofloxacin was observed to significantly decrease from 99% to 43% and 35% in the presence of HCOOH (quencher for electrons) and KI (quencher for holes), respectively (Figure 7c). Hence, it was proven that the e^−^ and h^+^ have a pivotal role to play in the degradation of levofloxacin. The formation of hydroxyl radicals in the photocatalytic reaction was determined by the TPA fluorescence process. TPA has the tendency to transform itself into a fluorescent compound, i.e., 2-hydroxyterephthalic acid, on encountering photogenerated ^•^OH. Therefore, it is presumed that the production of hydroxyl radical species in the system was proportional to the PL emission intensity of 2-hydroxyterephthalic acid. It can be seen in Figure 7d that the intensity of PL emission progressively rose under solar light irradiation with Z-scheme photocatalyst. Hence, it is suggested that hydroxyl radicals were formed throughout the production of 2-hydroxyterephthalic acid, and thus have a key role in the degradation of levofloxacin. This compound was also observed to support the proposed mechanism of the photocatalytic process. Figure 8 presents a diagrammatic illustration of the photocatalytic degradation mechanism of levofloxacin with Bi_2_WO_6_/C-dots/TiO_2_ under sunlight irradiation.

### 3.4. Investigation of Degradation Intermediates Formed in the Photocatalytic Degradation of Levofloxacin

**Figure 9:**
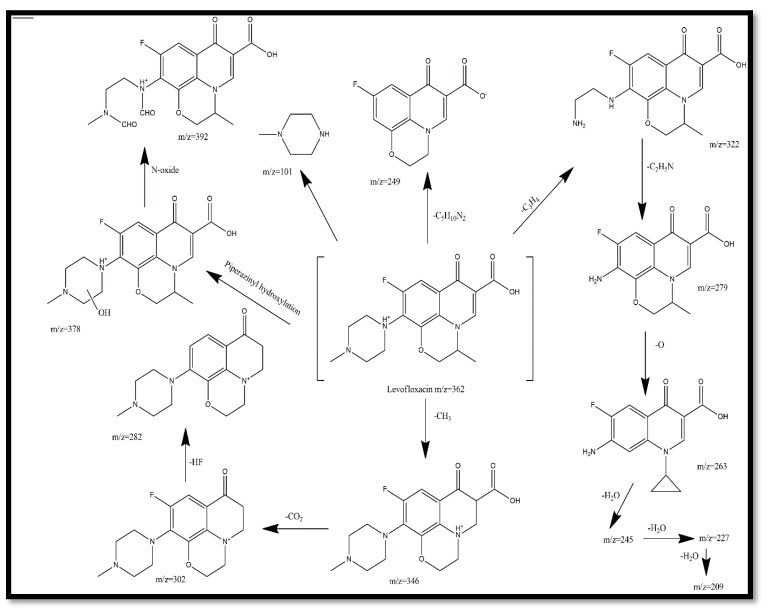
Identification of degradation intermediates formed during the photocatalytic reaction.

Attempts were made to elucidate the reaction intermediates composed during and after the photocatalytic degradation of levofloxacin at different time intervals and the plausible degradation pathway was constructed through the identification of the levofloxacin and its derivatives or intermediates from LC-MS analysis based on the consideration of molecular ion peaks [M + H]^+^ as shown in Figure 9. Levofloxacin has the molecular formula (M = 361.38 g/mol), according to its chemical formula C_18_H_20_FN_3_O_4_, and exhibits a protonated molecular peak at *m*/*z* = 362 [33]. In addition to the protonated peak of lexofloxacin, fewer intermediates were observed in the MS spectra of the levofloxacin samples withdrawn at time periods with the protonated peaks at *m*/*z* = 392, 249, 282, 209 and 101, respectively. The mass spectrograms are provided in Figure S3 of the Supplementary Information. The ring-opening or de-methylation at the N1 and N4 position, followed by further oxidation via hydroxyl radicals, leads to the formation of intermediate at *m*/*z* = 392 [51]. A fragment at *m*/*z* = 346 might have originated from the levofloxacin due to the loss of the methyl group (-CH_3_). The as-formed fragment underwent decarboxylation (-CO_2_), leading possibly to the formation of another intermediate with an *m*/*z* of 302. Consequently, with the loss of HF from the former intermediate, another mass fragment at *m*/*z* = 282 was obtained. A further *m*/*z* = 249 (intermediate) was formed when an intermediate with *m*/*z* = 346 underwent a loss of moiety C_5_H_10_N_2_. Likewise, another intermediate at *m*/*z* = 263 might be obtained by the ring-opening of the morpholine moiety at *m*/*z* = 279 and underwent dehydration with loss of H_2_O, revealed by the presence of the intermediate at *m*/*z* = 209 [54]. N-methyl piperazine with *m*/*z* value of 101 was derived from the levofloxacin with the cleavage of piperazinyl substituent [50].

The kinetics of the photocatalytic degradation of levofloxacin was studied using the Langmuir Hinshelwood kinetic model using the equation given below:

(6) $$\frac{dC}{dt}=\frac{k\times K_{C}}{\left(1+K_{C}\right)}$$

where *C* is the concentration at any time; *k* and *K_C_* are the limiting rate constants of reaction at maximum coverage under the given experimental conditions and equilibrium constant.

When the concentration of substrate was much less than 1, the above equation can be simplified to a pseudo-first-order kinetic equation as given below:

(7) $$ln\frac{C_{0}}{C}=kt$$

where *C*_0_ and *C* are the initial and final concentrations of the drug solution, *k* is the rate constant and can be calculated from the slope of the reaction, and *t* is the time interval of the photocatalytic reaction. 

**Figure 10:**
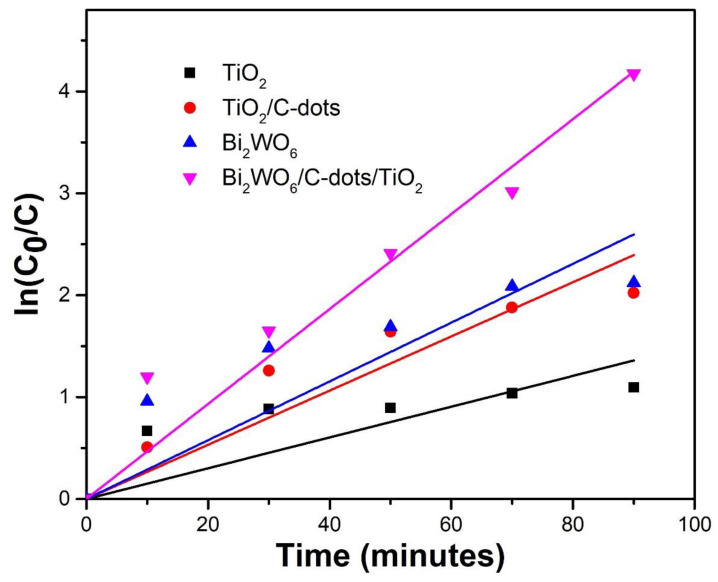
Graph of *ln*(*C*_0_/*C*) against time depicting the kinetics of photocatalytic reaction.

The degradation of the drug obeyed pseudo-first-order kinetics with all photocatalysts, but with different values of co-relation values (Figure 10). Other significant details are shown in tabular form (Table 2).

## 4. Conclusions

A novel ternary Z-scheme catalyst Bi_2_WO_6_/C-dots/TiO_2_ was successfully fabricated by an ultrasonic assisted hydrothermal method and characterized by several techniques. The crystallite size of the catalyst was found to be 34.29 nm from powder XRD studies. FESEM images explained that Bi_2_WO_6_ exhibited nanoflakes like morphology that aggregated to form nanosheets, while nanoparticles of TiO_2_/C-dots were found to be assembled over the surface of nanoflakes. PL studies emphasized the optical properties of Bi_2_WO_6_/C-dots/TiO_2_, indicating the hindrance in the recombination of photo-induced charge transporters in Z-scheme catalyst over bare Bi_2_WO_6_, suggesting that Z-scheme catalyst could enhance the photocatalytic activity. This hypothesis is supported by UV-DRS studies, which showed that the Z-scheme catalyst has a lower band gap, i.e., 2.50 eV compared to Bi_2_WO_6_ (2.75 eV), TiO_2_/C-dots (2.85 eV) and TiO_2_ (2.95 eV). The photocatalytic behavior of the synthesized catalysts was investigated by monitoring the degradation of fluoroquinolone levofloxacin drug under 90 min of sunlight illumination. Almost complete degradation was observed with Bi_2_WO_6_/C-dots/TiO_2_, whereas 88.3% and 87.24% degradation with Bi_2_WO_6_ and TiO_2_/C-dots under similar reaction conditions. Additionally, with respect to UV-DRS results, it could be concluded that Bi_2_WO_6_/C-dots/TiO_2_ can be considered as efficient potential photocatalyst in the visible region of the solar spectrum. The higher specific surface area of Bi_2_WO_6_/C-dots/TiO_2_ compared to bare Bi_2_WO_6_ calculated from nitrogen adsorption–desorption isotherm, also showed that the Z-scheme catalyst would provide more surface for the adsorption of the drug molecules, hence providing improved photocatalytic activity. Experimental parameters, including catalyst dose, reaction time, pH of drug solution and initial drug concentration were studied to optimize the photocatalytic reaction. A plausible mechanism for the degradation of the drug was also proposed based on scavenger radical trap experiments and TPA fluorescence technique. LC-MS analysis showed intermediates and other products at different stages of the photocatalytic process. 

## Figures and Tables

**Table 1:** Elemental composition of Bi_2_WO_6_/C-dots/TiO_2._

Element	Series	Unn. C [wt.%]	Norm. C [wt.%]	Atom. C [wt.%]	Error (3 Sigma) [wt.%]
Bismuth	M-Series	17.10	14.74	5.10	1.98
Carbon	K-Series	6.61	5.70	34.33	4.22
Oxygen	K-Series	1.45	1.25	5.65	1.47
Tungsten	L-Series	65.80	56.73	22.32	7.86
Titanium	K-Series	25.03	21.58	32.60	2.37
	Total	115.99	100.00	100.00	

**Table 2:** Kinetics data of photocatalytic reaction of levofloxacin with different catalysts.

S. No.	Photocatalyst	Rate Constant (k) s^−1^	R^2^
1	Bi_2_WO_6_/C-dots/TiO_2_	0.0007765	0.98797
2	Bi_2_WO_6_	0.0004808	0.97777
3	TiO_2_/C-dots	0.000443	0.95016
4	TiO_2_	0.00025183	0.96788

## Supplementary Materials

The following are available online at https://www.mdpi.com/2079-4991/10/5/910/s1, Figure S1: (a–c) FESEM images of Bi_2_WO_6_ at different magnifications. (d) EDS spectrum of Bi_2_WO_6_; Figure S2: TEM image of TiO_2_/C-dots; Figure S3: Mass spectrographs of levofloxacin drug at different time intervals of photocatalytic reaction; Table S1: Elemental composition of Bi_2_WO_6_.
