# Supplementary material for: Bi2WO6/C-Dots/TiO2: A Novel Z-Scheme Photocatalyst for the Degradation of Fluoroquinolone Levofloxacin from Aqueous Medium
Source: Nanomaterials (Basel). 2020 May 8;10(5):910. doi: 10.3390/nano10050910 (PMC7279409; doi:10.3390/nano10050910)
Supplement: Supplementary file 1 [file nanomaterials-10-00910-s001.pdf]

nanomaterials

MDPI

Article

Bi_2WO_6/C-Dots/TiO_2: A Novel Z-Scheme Photocatalyst for the Degradation of Fluoroquinolone Levofloxacin from Aqueous Medium

Shelja Sharma [1], Alex O. Ibhadon [2], M. Grazia Francesconi [3], Surinder Kumar Mehta [4], Sasikumar Elumalai [1], Sushil Kumar Kansal [5,*], Ahmad Umar [6,*] and S. Baskoutas [7]

1 Chemical Engineering Division, Centre of Innovative and Applied Bioprocessing, Mohali-140306, India; sshhelja@yahoo.in (S.S.); sasikumar@ciab.res.in (S.E.)
2 Department of Chemical Engineering, The University of Hull, Hull HU6 7RX, United Kingdom; a.o.ibhadon@hull.ac.uk
3 Department of Chemistry, University of Hull, Hull HU6 7RX, United Kingdom; m.g.francesconi@hull.ac.uk
4 Department of Chemistry and Centre of Advanced Studies, Panjab University, Chandigarh 160014, India; skmehta@pu.ac.in
5 Dr. S.S. Bhatnagar University Institute of Chemical Engineering and Technology, Panjab University, Chandigarh 160014, India
6 Department of Chemistry, Faculty of Science and Arts, Promising Centre for Sensors and Electronic Devices (PCSED), Najran University, Najran-11001, Kingdom of Saudi Arabia
7 Department of Materials Science, University of Patras, Patras-26504, Rio Achaia, Greece; bask@upatras.gr
* Correspondence: sushilkk1@pu.ac.in (S.K.K.); ahmadumar786@gmail.com (A.U.)

SUPPORTING INFORMATION

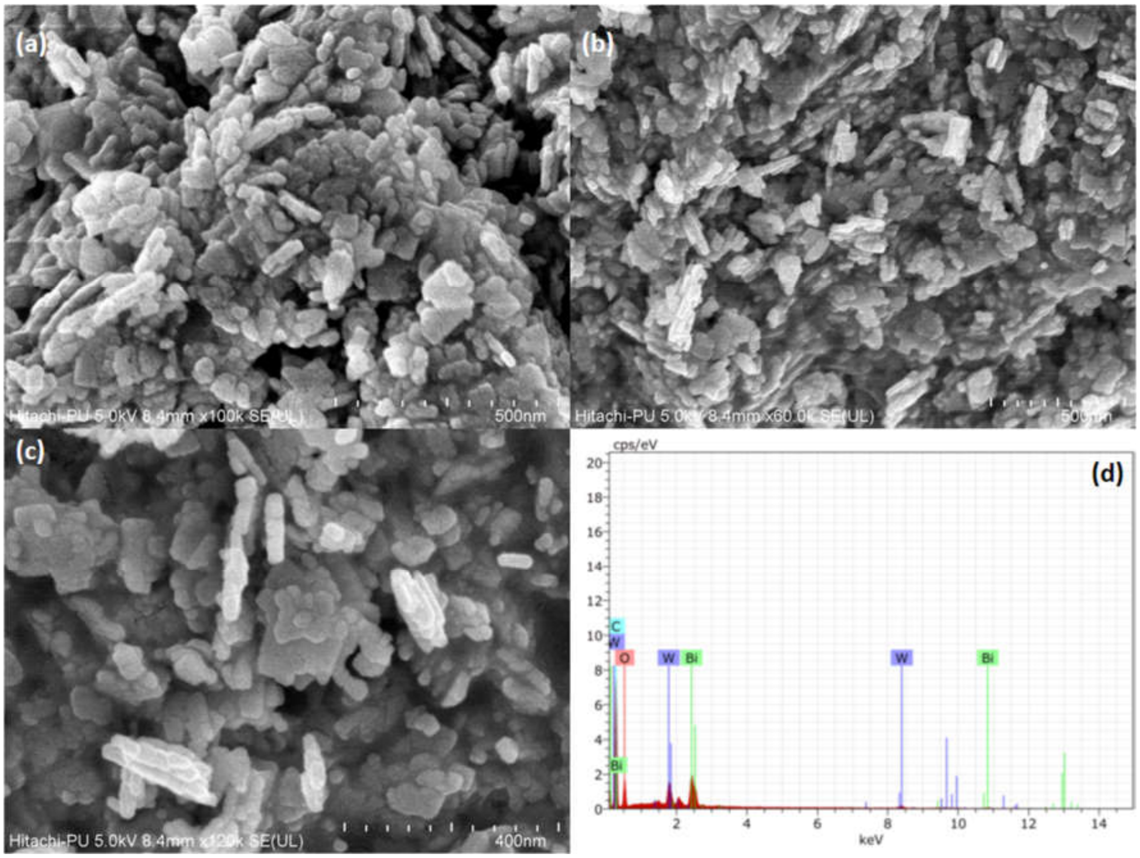

Figure S1. (a,b,c) FESEM images of Bi_2WO_6 at different magnifications. (d) EDS spectrum of Bi_2WO_6.

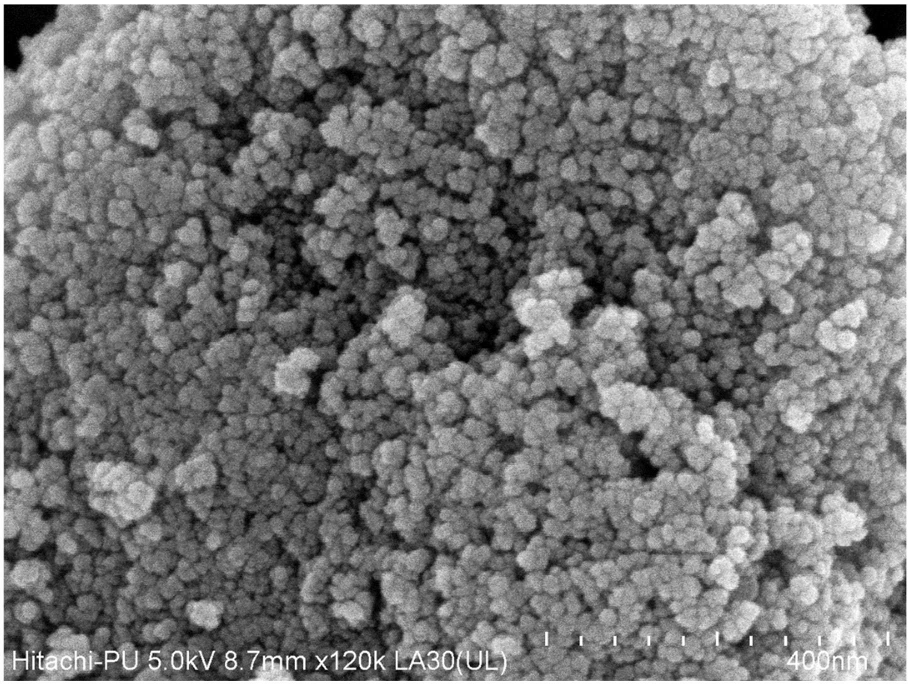

Figure S2. FESEM image of TiO_2/C-dots.

Table S1. Elemental composition of Bi_2WO_6.

Element	Series	unn. C [wt.%]	norm. C [wt.%]	Atom. C [wt.%]	Error (3 Sigma) [wt.%]
Oxygen	K-Series	14.21	12.75	17.69	6.67
Bismuth	M-Series	20.11	18.05	1.92	2.26
Tungsten	L-Series	30.60	27.47	3.32	4.25
Carbon	K-Series	46.48	41.72	77.08	17.70
	Total	111.40	100.00	100.00	

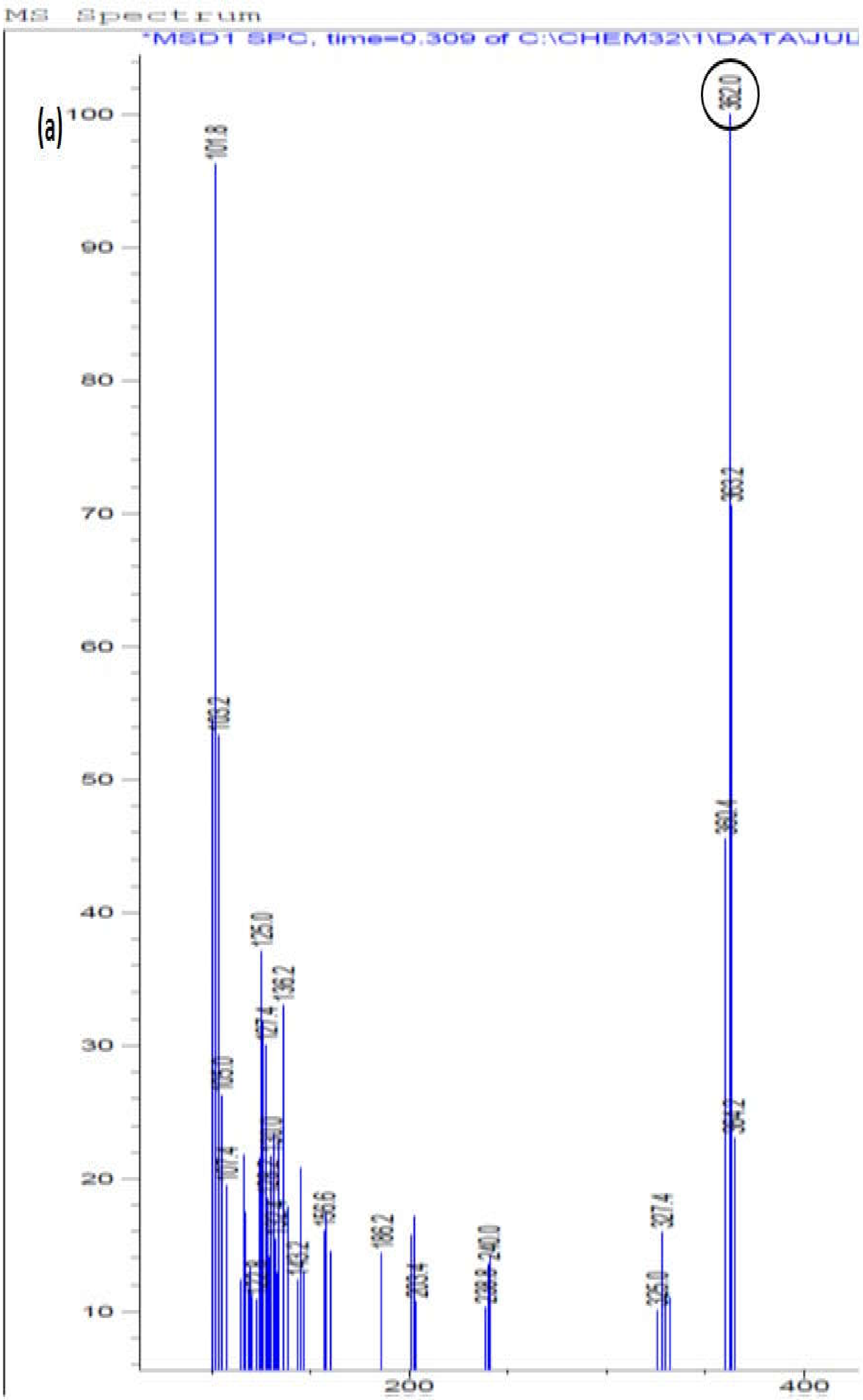
MS Spectrum
*MSD1 SPC, time=0.309 of C:\CHEM32\1\DATA\JUL
(a)
100
90
80
70
60
50
40
30
20
10
200
400
362.0
101.8
363.2
103.2
360.4
125.0
136.2
127.4
364.2
327.4
156.6
186.2
240.0
325.0

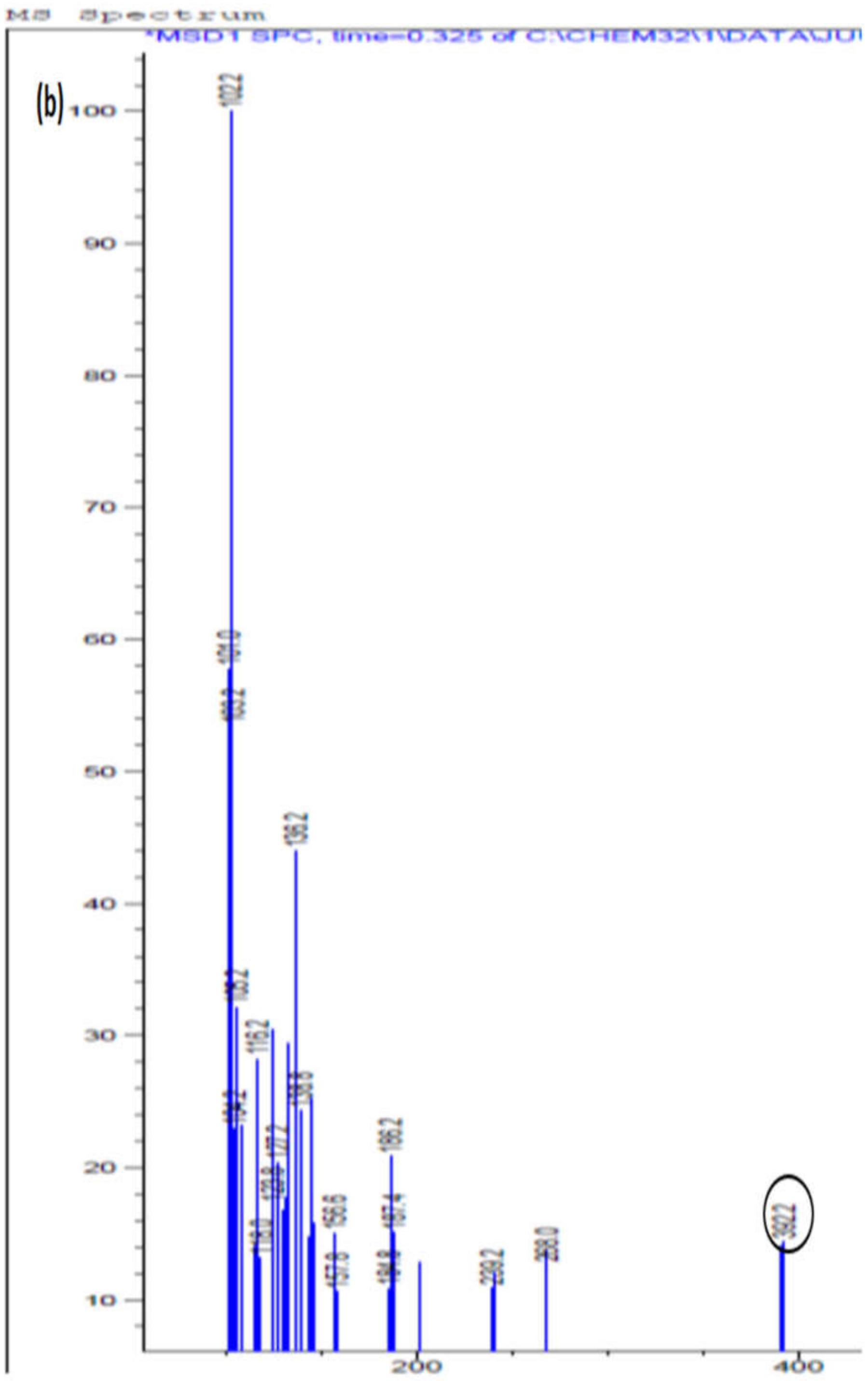
MS Spectrum
*MSD1 SPC, time=0.325 of C:\CHEM32\1\DATA\JU
(b)
100
90
80
70
60
50
40
30
20
10
200
400
102.2
101.0
136.2
116.2
186.2
156.6
187.4
239.2
268.0
392.2

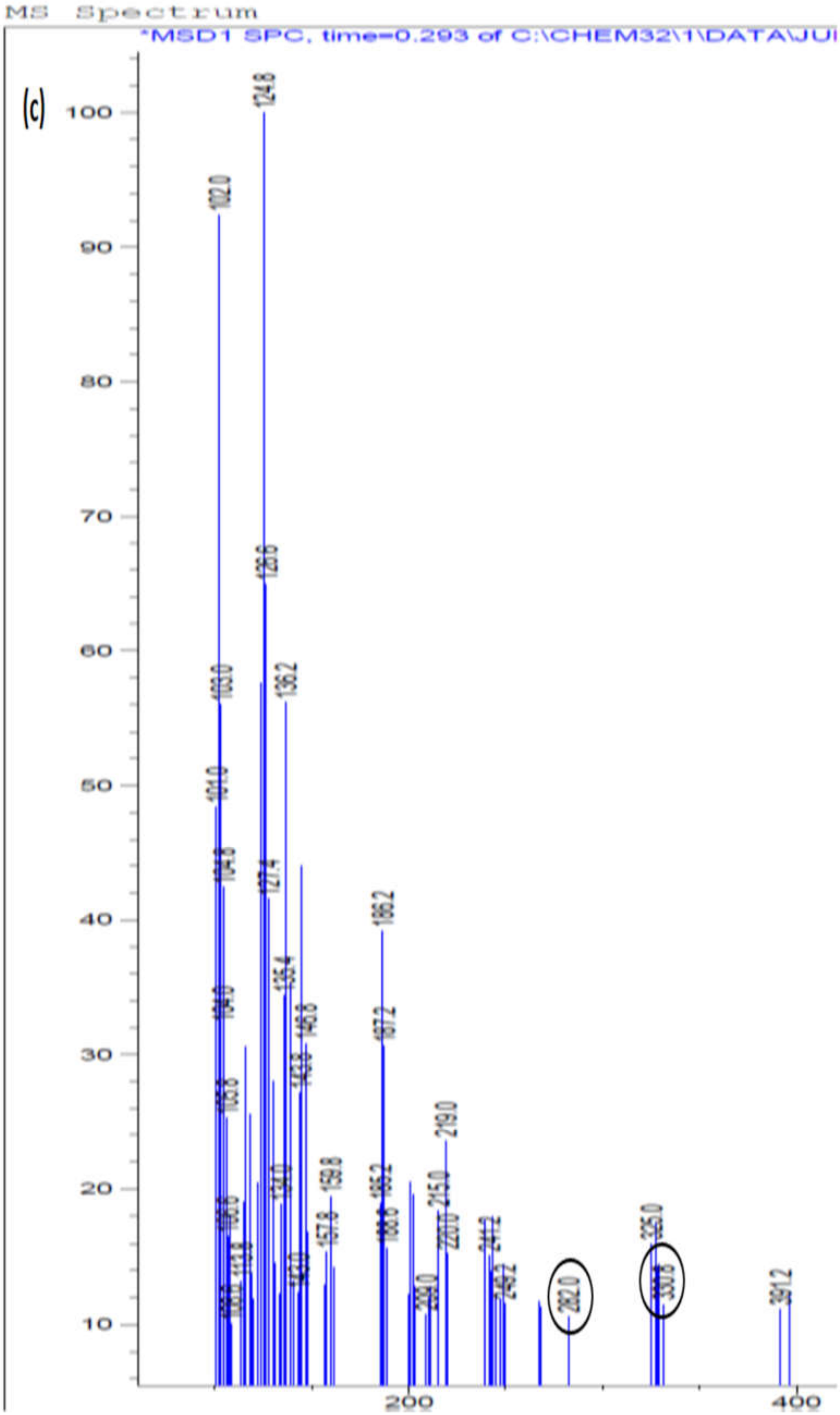
MS Spectrum
*MSD1 SPC, time=0.293 of C:\CHEM32\1\DATA\JU
(c)
100
90
80
70
60
50
40
30
20
10
200
400
124.8
102.0
126.6
103.0
136.2
101.0
127.4
186.2
135.4
187.2
219.0
159.8
215.0
335.0
282.0
391.2

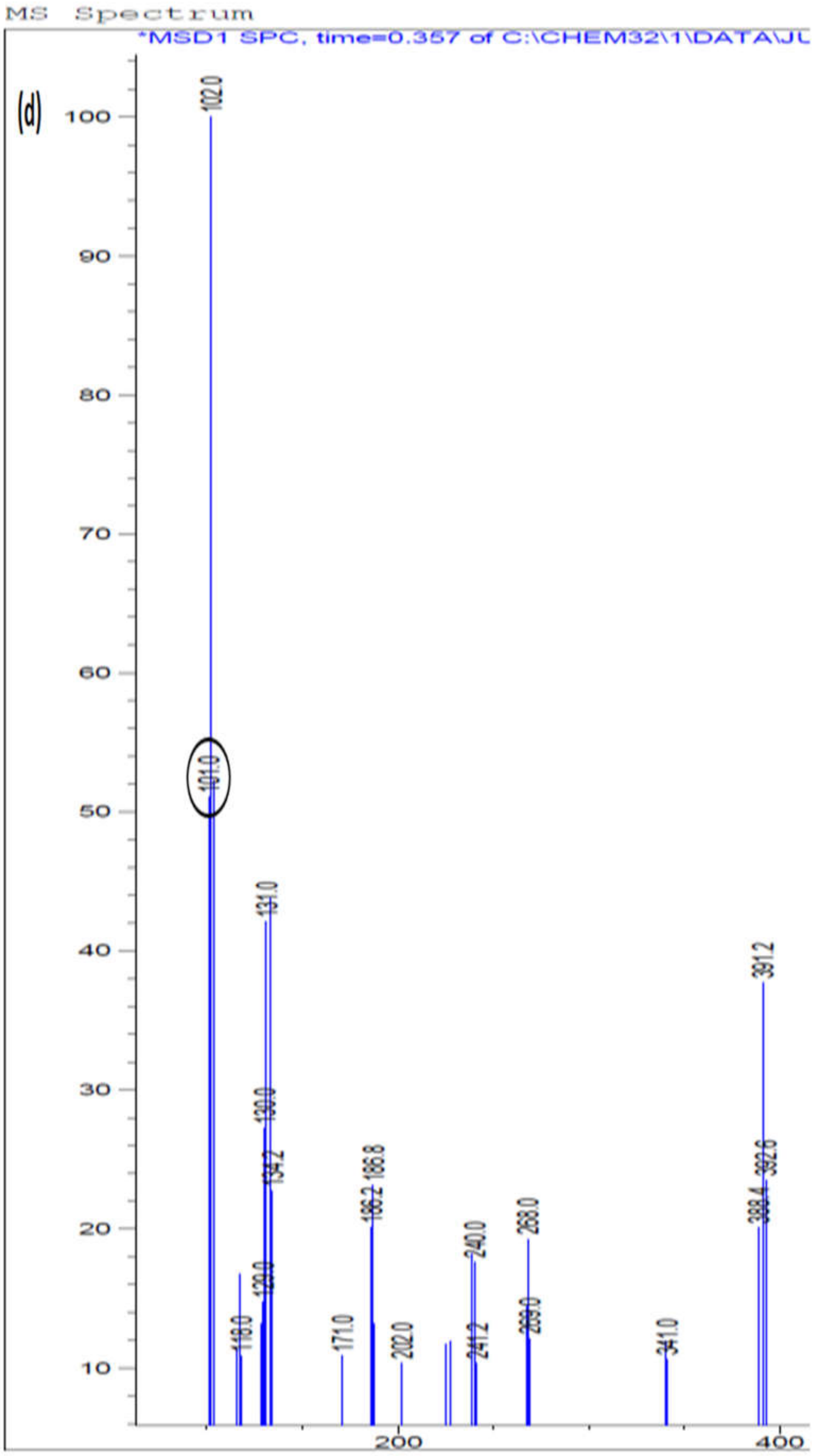
MS Spectrum
*MSD1 SPC, time=0.357 of C:\CHEM32\1\DATA\JL
(d)
100
90
80
70
60
50
40
30
20
10
200
400
102.0
101.0
131.0
130.0
134.2
129.0
118.0
171.0
186.8
186.2
202.0
240.0
241.2
268.0
269.0
341.0
391.2
392.6
388.4

Figure S3. MS spectrum of [M+H]+ ions of levofloxacin (a) parent compound; (b-d) its main products formed during the solar light induced photocatalytic degradation.
